# Population pharmacokinetic/pharmacodynamic modelling to evaluate favipiravir in combination with lopinavir–ritonavir in patients with COVID‐19

**DOI:** 10.1002/bcp.70507

**Published:** 2026-03-23

**Authors:** Akosua A. Agyeman, Laura Buggiotti, Li‐An K. Brown, Jose A. Guerra‐Assuncao, Japhette E. Kembou‐Ringert, Wen Y. Mak, Judith Breuer, David M. Lowe, Joseph F. Standing, FLARE Investigators

**Affiliations:** ^1^ Infection, Immunity and Inflammation Research and Teaching Department, Great Ormond Street Institute of Child Health University College London London UK; ^2^ Institute of Immunity and Transplantation University College London, Royal Free Campus London UK; ^3^ Clinical Research Centre Penang General Hospital George Town Penang Malaysia; ^4^ Institute for Clinical Research National Institutes of Health Shah Alam Selangor Malaysia; ^5^ Department of Microbiology Great Ormond Street Hospital for Children London UK; ^6^ Department of Clinical Immunology Royal Free London NHS Foundation Trust London UK; ^7^ Department of Pharmacy Great Ormond Street Hospital for Children London UK

**Keywords:** COVID‐19, favipiravir, lopinavir–ritonavir, population pharmacokinetic/pharmacodynamic modelling, viral whole‐genome sequence

## Abstract

**Aims:**

The repurposed use of favipiravir in COVID‐19 has been reported to have limited clinical efficacy, yet it has been widely used in some countries. Favipiravir causes mutagenesis in RNA viruses, and it is currently unknown whether it may have a measurable effect on the virus in humans. In this study, we report the secondary endpoints from the phase 2a randomized, double‐blind, 2 × 2 factorial placebo‐controlled FLARE trial, which studied the combination of favipiravir and lopinavir–ritonavir.

**Methods:**

Population pharmacokinetic and viral dynamic models including data from 59 and 235 participants respectively were developed to assess the drug effect of favipiravir in combination with lopinavir–ritonavir. Viral whole‐genome sequencing was performed.

**Results:**

The final joint model consisted of a slope–intercept exponential decay viral dynamic model and a one‐compartment model with first‐order absorption and elimination for favipiravir pharmacokinetics. The mean death rate of infected cells (*δ*) was 0.96 day^−1^. Favipiravir mean apparent clearance (*CL*/*F*) and mean apparent volume of distribution were 1.73 L/h and 22.1 L, respectively. Favipiravir co‐administration with lopinavir–ritonavir increased *CL*/*F* by twofold, representing an unexpected drug–drug interaction. Favipiravir and lopinavir–ritonavir drug effects on *δ* did not improve model fit in the final joint model, indicating that neither drug has any antiviral effect against SARS‐CoV‐2. Viral sequencing did not show a clear mutagenic signature.

**Conclusions:**

Our findings suggest that favipiravir does not have antiviral activity at the dose used in our study, and further work to understand the mechanistic basis of the drug–drug interaction with lopinavir–ritonavir is required.

**What is already known about this subject**

- Our intention‐to‐treat analysis showed that neither favipiravir, lopinavir–ritonavir or their combination significantly reduced viral load, but this analysis assumed perfect treatment adherence not accounting for interindividual variability.
- Population pharmacokinetic/pharmacodynamic (PK/PD) modelling and viral whole‐genome sequencing studies to ascertain the observed variability in favipiravir pharmacokinetics and viral dynamics are scarce.

**What this study adds**

- Co‐administration of favipiravir with lopinavir–ritonavir causes about a two‐fold increase in apparent clearance of favipiravir during early treatment of COVID‐19, representing an unexpected new drug–drug interaction.
- PK/PD modelling showed no significant effect of favipiravir and lopinavir–ritonavir on the death rate of infected cells.
- No genetic mutagenic signature of favipiravir was seen.

## 1 INTRODUCTION

The COVID‐19 pandemic initiated a broad range of therapeutic studies including vaccines, monoclonal antibodies, and novel and repurposed antivirals.^1^ Despite efforts to find effective treatments and preventive strategies, there remain unprecedented challenges. For example, although vaccinations have reduced the rate of infections, hospitalization and deaths, the emergence of SARS‐CoV‐2 variants and sub‐variants continues to hamper the duration of protection. Despite the reported efficacy of novel oral antiviral agents such as molnupiravir and nirmatrelvir/ritonavir,^2^, ^3^, ^4^, ^5^ viral rebound, failure to clear the virus after a 5‐day course and viral mutagenesis caused by antivirals have been reported.^6^, ^7^, ^8^ In addition, novel agents are expensive, making them less accessible in low‐ and middle‐income countries.

Therefore, repurposing of existing drugs to treat COVID‐19 disease continues to be of interest. Most repurposed drugs that have been studied have not shown significant efficacy either as single agents or in combination therapy,^9^ but many of these studies did not take a detailed clinical pharmacology approach, meaning the mechanistic basis for trial failure was sometimes uncertain.^10^

Favipiravir is a broad‐spectrum antiviral against a range of RNA viruses, and early reports suggested that it may have had anti‐SARS‐CoV‐2 activity at the upper end of the clinically achievable concentration range.^11^ As an RNA‐dependent RNA polymerase (RdRp) inhibitor, favipiravir can be incorporated into viral RNA, causing the accumulation of deleterious mutations or chain termination. RdRp inhibitors therefore cause a characteristic viral genomic signature that can be observed following clinical treatment.^8^

In September 2020, we opened the FLARE trial (ClinicalTrials.gov: NCT04499677) to recruitment. Expecting favipiravir alone to lack sufficient antiviral effects, we added the only available protease inhibitor, lopinavir–ritonavir, hoping the combination of agents with modest activity might have additive or synergistic clinical effects. FLARE was a phase 2a randomized, double‐blind, 2 × 2 factorial placebo‐controlled trial of favipiravir, lopinavir–ritonavir or their combination for early treatment of COVID‐19.^12^ In addition to viral load at Day 7, we collected detailed secondary endpoints including pharmacokinetics and daily viral loads and performed longitudinal viral whole‐genome sequencing. The intention‐to‐treat analysis of the FLARE trial found that no treatment significantly reduced viral load at the studied doses, and the co‐administration of lopinavir–ritonavir resulted in lower favipiravir plasma concentrations. Other studies on favipiravir have shown mixed clinical efficacy results,^13^, ^14^, ^15^ and few studies have reported on pharmacokinetic (PK) assessment.^16^, ^17^ To our knowledge, however, a joint population PK/pharmacodynamic (PD) model has not yet been reported for favipiravir alone or in combination with other agents for COVID‐19, and little is known about its viral mutagenic effects in SARS‐CoV‐2.

Longitudinal viral dynamic modelling has the potential to uncover small viral load changes that would not be seen by looking at a single time point.^18^ This report therefore details the following secondary and exploratory endpoints: rate of viral load decline, PK/PD analysis of favipiravir, and the proportion of participants with deleterious or resistance‐conferring mutations in SARS‐CoV‐2 viral loads in the ‘as‐treated’ population of the FLARE trial, accounting for actual dose history. Firstly, a viral dynamic model was developed to characterize the observed viral load trajectories. Secondly, a population PK model for favipiravir was developed. Thirdly, a joint population PK/PD model was developed to ascertain the magnitude of favipiravir and lopinavir–ritonavir drug effects on the death rate of infected cells. Finally, viral whole‐genome sequencing was performed to evaluate the treatment effect on viral mutagenesis.

## 2 METHODS

### 2.1 Study participants, viral load assessment and favipiravir concentration measurement

Participants were ambulatory outpatients aged between 18 and 70 years who had either developed symptoms of COVID‐19 within the last 5 days or had tested positive for SARS‐CoV‐2 by polymerase chain reaction (PCR) and were within 7 days of symptom onset or who were asymptomatic but had tested positive by PCR within the previous 48 h. Participants were randomized to receive one of the following treatments: favipiravir (1800 mg twice daily on Day 1, followed by 400 mg four times daily from Day 2 to Day 7) plus lopinavir–ritonavir (400/100 mg twice daily on Day 1, followed by 200/50 mg four times daily from Day 2 to Day 7), favipiravir plus lopinavir–ritonavir placebo, favipiravir placebo plus lopinavir–ritonavir or matched placebos of both drugs.

Daily viral loads were measured in saliva samples (collected into RNA preservation buffer [Saliva RNA Collection and Preservation Devices, Norgen Biotek, Canada]) by a quantitative PCR (qPCR) with a cycle threshold (Ct) value of 40 as the lower limit of quantification (LLOQ). Blood samples for renal function and liver function were taken at baseline and on Day 7 and processed in the diagnostic laboratory at Great Ormond Street Hospital for Children, London, UK. Blood samples for favipiravir trough (i.e., pre‐dose sample) and peak (i.e., post‐dose sample) concentration measurements were collected on Day 7. The post‐dose sample was collected 30–60 min after drug administration. After discarding 2 mL of blood from the cannula, samples consisted of 2 mL of whole blood collected in EDTA‐containing tubes. Plasma was separated by centrifugation (850 g, 15 min) with the virus inactivated by adding 4 mL of ethanol for each 1 mL of plasma. Following a further centrifugation step, samples were frozen at −20°C. Plasma favipiravir concentrations were quantified by the LSI Medience Corporation in Japan on behalf of FUJIFILM Toyama Chemical Co. with an LLOQ of 0.1 mg/L.

### 2.2 Viral whole‐genome sequencing

Viral whole‐genome sequencing was carried out by bait capture with biotinylated RNA oligonucleotides used in the Agilent SureSelectXT (SSXT) protocols. A design targeting SARS‐CoV‐2 was designed in‐house and synthesized by Agilent Technologies (available through the Agilent Community Designs [NGS] program as the SureSelect CD Pan Human Coronavirus Panel).

Briefly, double‐stranded cDNA was sheared using a Covaris E220 Focused‐Ultrasonicator System (42 s, Peak Incident Power [PIP] of 75, duty factor of 10 and cycles per burst of 1000). End‐repair, non‐templated addition of 3′ poly(A), adaptor ligation, pre‐capture PCR, hybridization, post‐capture PCR and all post‐reaction clean‐up steps were performed on the Bravo NGS Workstation Option B (Agilent Technologies) using the SureSelectXT Low Input Kit (Agilent Technologies), with minor modifications to the manufacturer's protocol to account for variable pathogen loads. Quality control steps were performed using the 4200 TapeStation (Agilent Technologies). Libraries were sequenced using the NextSeq 500 platform (Illumina), using a 150‐cycle kit set up as 75‐bp paired‐end.

### 2.3 Data analysis

The model development process was performed in three parts. Firstly, a viral dynamic model was developed to describe the observed viral load trajectories. Secondly, a population PK model was developed for favipiravir. Finally, the antiviral effect was evaluated via a joint population PK/PD model based on the viral dynamic and population PK models.

Nonlinear mixed‐effects modelling was performed using the R package nlmixr2 (Version 2.0.8) with first‐order conditional estimation (FOCE) and FOCE with interaction (FOCE‐I) methods applied as appropriate. Individual parameters were expressed on the logarithmic scale as shown below.

(1) $$\theta_{i}=\exp\left(\theta_{p}+\mathrm{IIV}\right)$$

where $\theta_{i}$ is the individual value, $\theta_{p}$ is the log‐transformed population typical value, and IIV is the interindividual variability assumed to be normally distributed with a mean of zero and variance of $\omega^{2}$.

#### 2.3.1 Viral dynamic model

Both simple (i.e., slope–intercept exponential decay [SI] and reduced target cell limited [rTCL]) and complex (i.e., target cell limited [TCL] and TCL with eclipse phase [TCLE]) models were fitted to viral load data to select the structural model that best characterized the observations. Briefly, for the SI model, elimination of viral particles (*V*) occurs at an overall rate of *δ*. In the rTCL model, *V* infect susceptible target cells at rate *β*, proportional to the fraction of uninfected target cells remaining (*f*). Virus is produced at a maximum rate constant of *γ* with an overall elimination rate of *δ*. For the TCL model, *V* infect uninfected target cells (*T*) at rate *β*, resulting in productively infected cells (*I*), which release viruses at rate *ρ* and are cleared at rate *c*. Productively infected cells die at a rate *δ*. In the TCLE model, *V* infect uninfected target cells (*T*) at rate *β* to produce latently infected cells (*I*_1_) during an incubation period and transition to productively infected cells (*I*_2_) at rate *k*. Productively infected cells (*I*_2_) release viruses at rate *ρ*, with clearance rate *c*. These productively infected cells die at rate *δ*. Initial conditions for *V*, *T*, infected cells ( *I*, *I*_*1*_ and *I*_*2*_) and *f* were 0.1 copies/mL, 1.33 × 10^5^ cells/mL, 0 cells/mL and 1, respectively.^19^, ^20^, ^21^ For SI and rTCL, *δ* translates as the overall viral elimination rate under the assumption of quasi‐steady state between *I* and *V* due to the typically faster *c* than *δ*.

Details of the SI, rTCL, TCL and TCLE models have been previously described.^22^

For the SI and rTCL models, time since symptom onset was used for model fitting, whilst time since infection was used for the TCL and TCLE models following a sensitivity analysis of the incubation period. Fixed incubation periods of 1–14 days in 1‐day increments were tested. Interindividual variability (IIV) for each estimated parameter followed a full covariance matrix structure, and an additive error model was used to describe the residual unexplained variability (RUV). Viral loads below the LLOQ were censored as per the M3 method.^23^

#### 2.3.2 Population PK model for favipiravir

One‐ and two‐compartment models with first‐order absorption and elimination and the inclusion of a transit compartment were evaluated. Bioavailability was fixed to unity. Time‐dependent clearance of favipiravir was considered, and the percentage increase in favipiravir apparent clearance (*CL*/*F*) per day since the start of the study was fixed to a previous literature‐derived value of 6.14%.^24^ The effect of body size on *CL*/*F* and apparent volume of distribution (*V*/*F*) was included in the base model via allometric scaling (weight centred at 70 kg) with fixed exponents of 0.75 and 1 for *CL*/*F* and *V*/*F* terms, respectively.^25^ IIV for absorption rate constant (*k*_*a*_), *CL*/*F* and *V*/*F* was specified individually and evaluated sequentially in the base model. The combined (additive and proportional) error model was initially used to model RUV.

Secondary PK parameters (i.e., maximum plasma concentration [*C*_max_], time to reach *C*_max_ [*T*_max_], minimum plasma concentration [*C*_trough_] and area under the concentration–time curve up to 6 h [AUC_0–6 h_]) following a single dose of 400‐mg favipiravir were determined based on the population mean PK estimates from the final model using the R package, rxode2 (Version 2.0.11).

#### 2.3.3 Covariate model

After the base model was established for the viral dynamic and PK models, the effects of covariates were explored. The effect of weight, sex, and age on baseline viral load and the death rate of infected cells (*δ*) was explored for the viral dynamic model. For the PK model, the influence of co‐administration of lopinavir–ritonavir, age, sex, uric acid, renal function (serum creatinine) and liver function (alanine transaminase, alkaline phosphatase, bilirubin and aspartate transaminase) was tested on *CL*/*F*. Covariates were introduced separately into the base model and retained if a statistically significant decrease in objective function value (OFV) of at least 3.84 (*p* < .05, chi‐squared distribution and 1 degree of freedom) was observed.^26^ Once all covariates were included, a backward elimination procedure was performed, and a covariate was retained if its removal resulted in a statistically significant increase in OFV greater than 6.63 (*p* < .01, chi‐squared distribution and 1 degree of freedom).^26^

Covariates were introduced into the model with the following equation:

(2) $$\theta_{i}=\exp\left(\theta_{p}+\mathrm{II}V+\mathrm{COV}*\text{COVeff}\right)$$

where COV is the log‐transformed covariate variable centred on the median value for continuous covariates. For categorical covariates, COV is coded as 1 in the presence of the covariate and 0 when absent. COVeff is the estimated covariate effect.

#### 2.3.4 Final population PK/PD model

**FIGURE 1:**
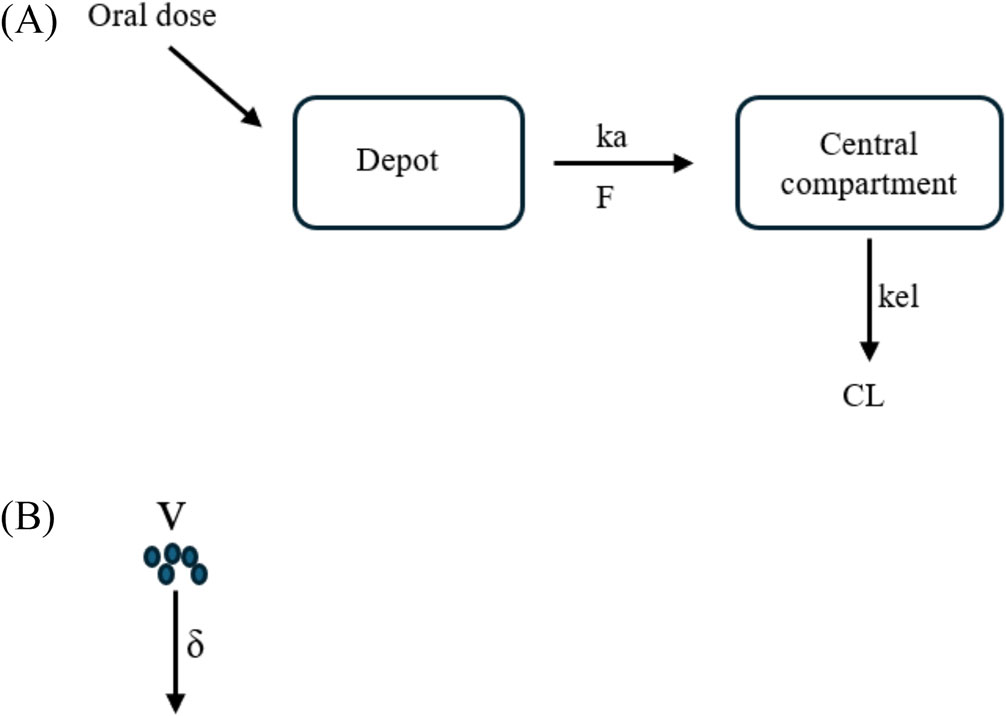
Schematic representation of the final population PK/PD model. (A) A one‐compartment pharmacokinetic model with first‐order absorption and elimination. (B) Slope–intercept exponential decay viral dynamic model. *CL*, clearance; *F*, bioavailability; *k*_*a*_, absorption rate constant; *k*_*el*_, elimination rate constant; *V*, viral particles; *δ*, overall viral elimination rate.

The individual PK parameters (*k*_*a*_, *V*/*F* and *CL*/*F*) from the final population PK model for favipiravir were integrated into the selected final viral dynamic model to develop a joint PK/PD model (Figure 1). Lopinavir–ritonavir PK parameters were fixed to literature‐derived values.^27^ Antiviral effect was modelled as a stimulatory function on *δ* and included in the joint model as follows:

(3) $$1+\left(\text{beta}\times \mathrm{cp}\right)$$

where beta is the parameter associated with the positive relationship between plasma drug concentration (*cp*) and *δ*. RUV for viral load data was modelled by an additive error model.

#### 2.3.5 Model evaluation

Models were assessed based on significant reduction in OFV as described in the covariate model section above for nested models with one degree of freedom. Otherwise, the lowest Bayesian information criterion (BIC) and Akaike information criterion (AIC) informed model choice. Additionally, the precision and biological plausibility of parameter estimates, visual predictive check (VPC) and goodness‐of‐fit (GOF) plots informed model selection. A bootstrap procedure of 200 runs was also conducted to evaluate the robustness of the final joint PK/PD model.

#### 2.3.6 Sequence analysis

The raw fastq reads were adapter‐trimmed, and low‐quality reads were removed using the fastp algorithm. The reads were aligned against the Wuhan‐Hu‐1 reference genome (NC_045512.2, EPI_ISL_402125). Consensus sequences were then called at a minimum of 30× coverage. The entire processing of raw reads to consensus was carried out using the nf‐core/viralrecon pipeline.^28^ Only samples producing genomes with at least 90% genome coverage at 10× sequencing depth were kept for further analysis. Variant calling was performed using the FreeBayes algorithm.^29^

### 2.4 Nomenclature of targets and ligands

Key protein targets and ligands in this article are hyperlinked to corresponding entries in http://www.guidetopharmacology.org and are permanently archived in the Concise Guide to PHARMACOLOGY 2021/22.^30^

## 3 RESULTS

**FIGURE 2:**
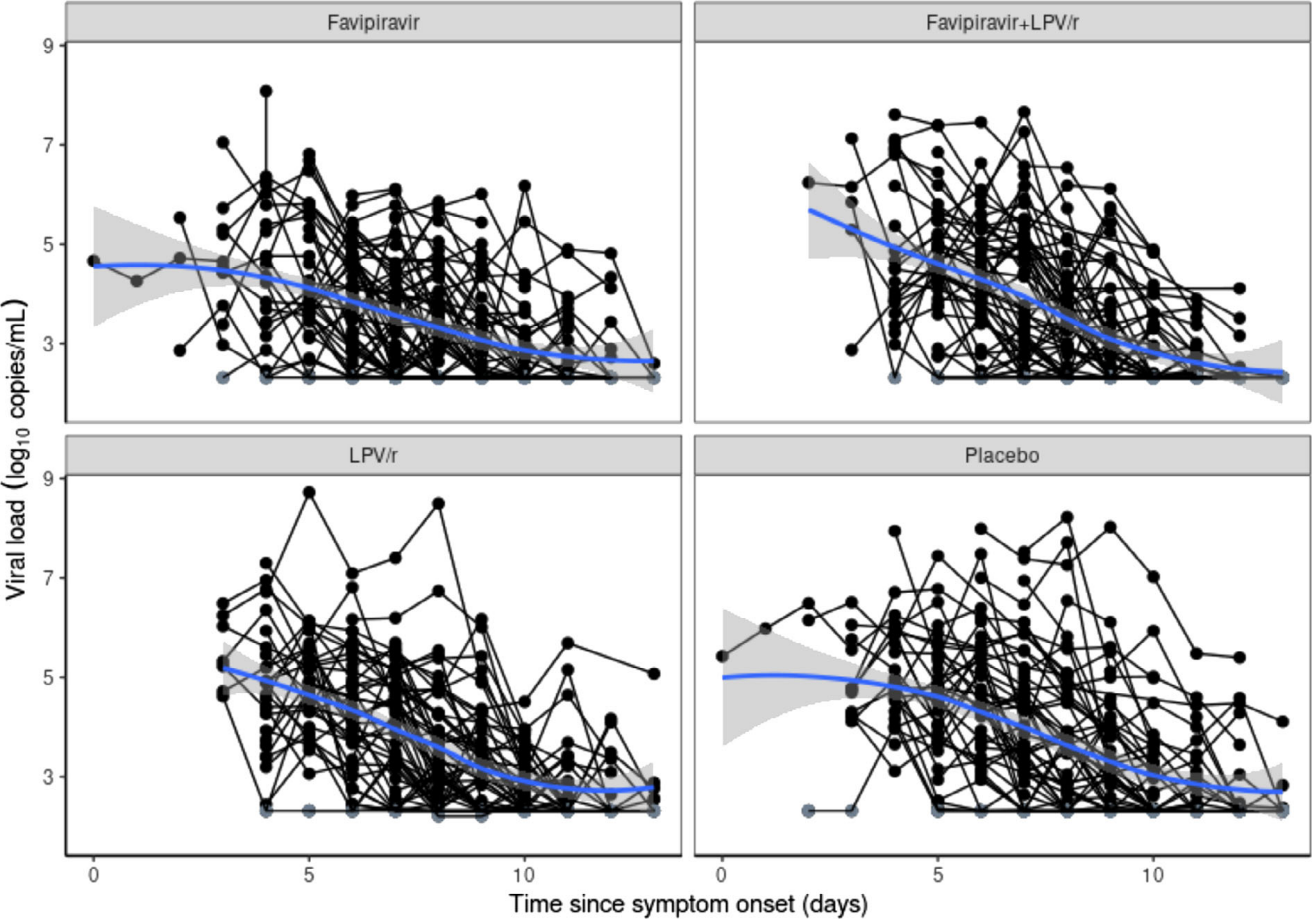
Longitudinal viral load data stratified by treatment arms in association with symptom onset. Black dots are data points. Grey dots are data points below the lower limit of quantification. Blue lines represent loess smoothing through the data with a 95% confidence interval shown as grey shading. LPV/r, lopinavir–ritonavir.

**FIGURE 3:**
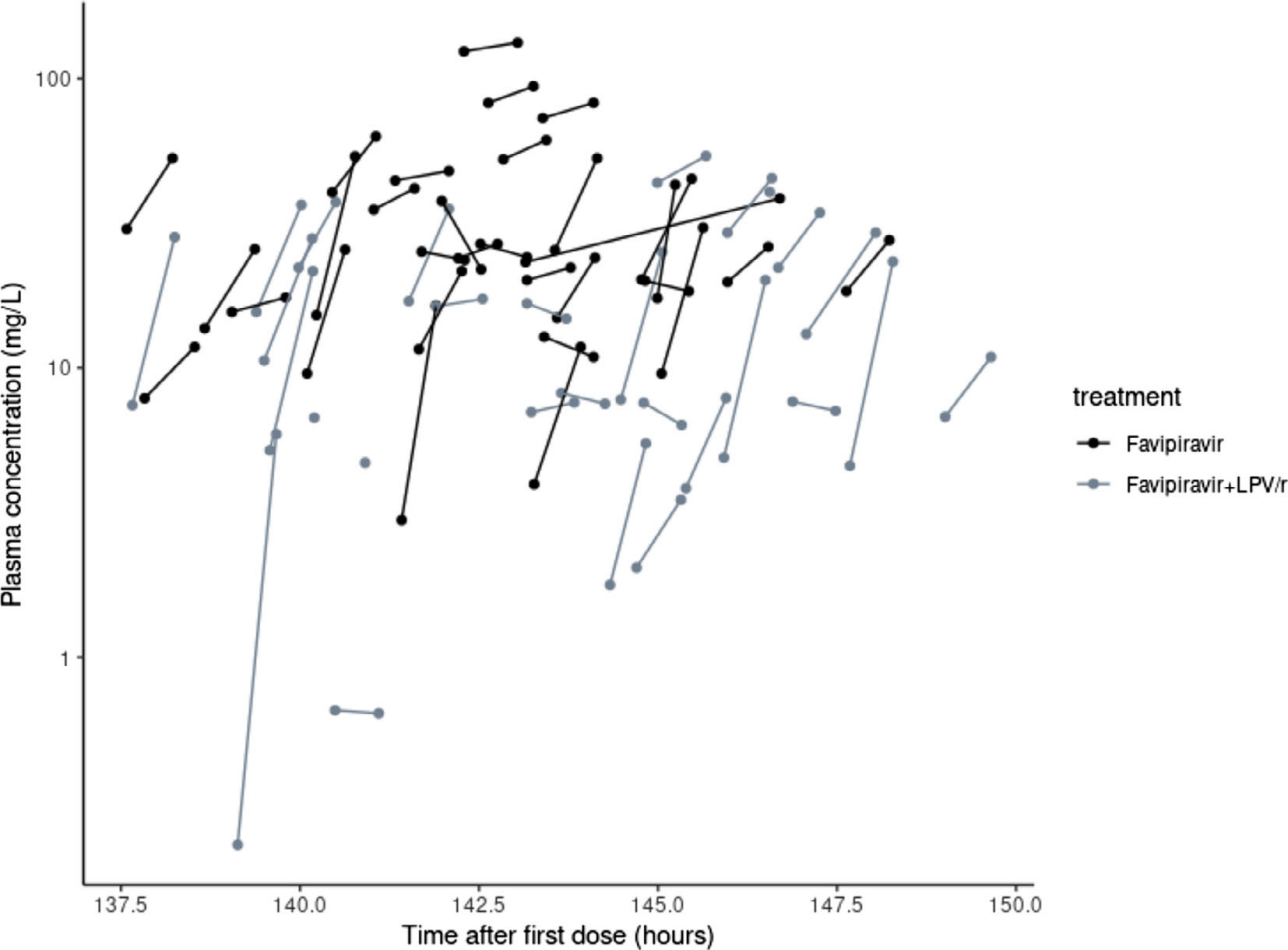
Favipiravir plasma concentrations following early treatment with favipiravir alone or in combination with lopinavir–ritonavir (LPV/r).

**FIGURE 4:**
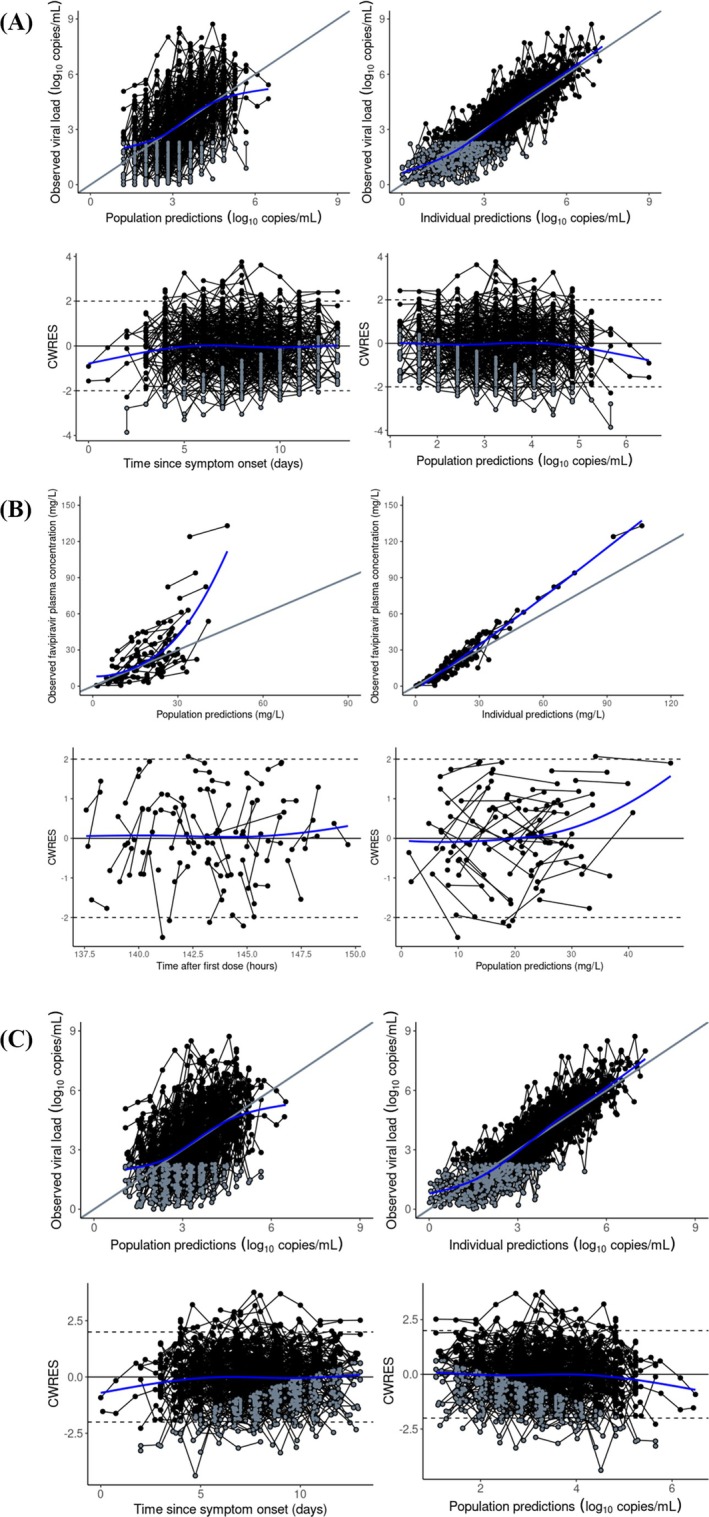
Goodness‐of‐fit plots for (A) slope–intercept exponential decay viral dynamic model, (B) population pharmacokinetic model for favipiravir and (C) final population PK/PD model. Black dots are data points. Grey dots are data points below the lower limit of quantification. The grey line indicates the line of identity. Blue lines represent loess smoothing through the data. CWRES, conditional weighted residuals.

**FIGURE 5:**
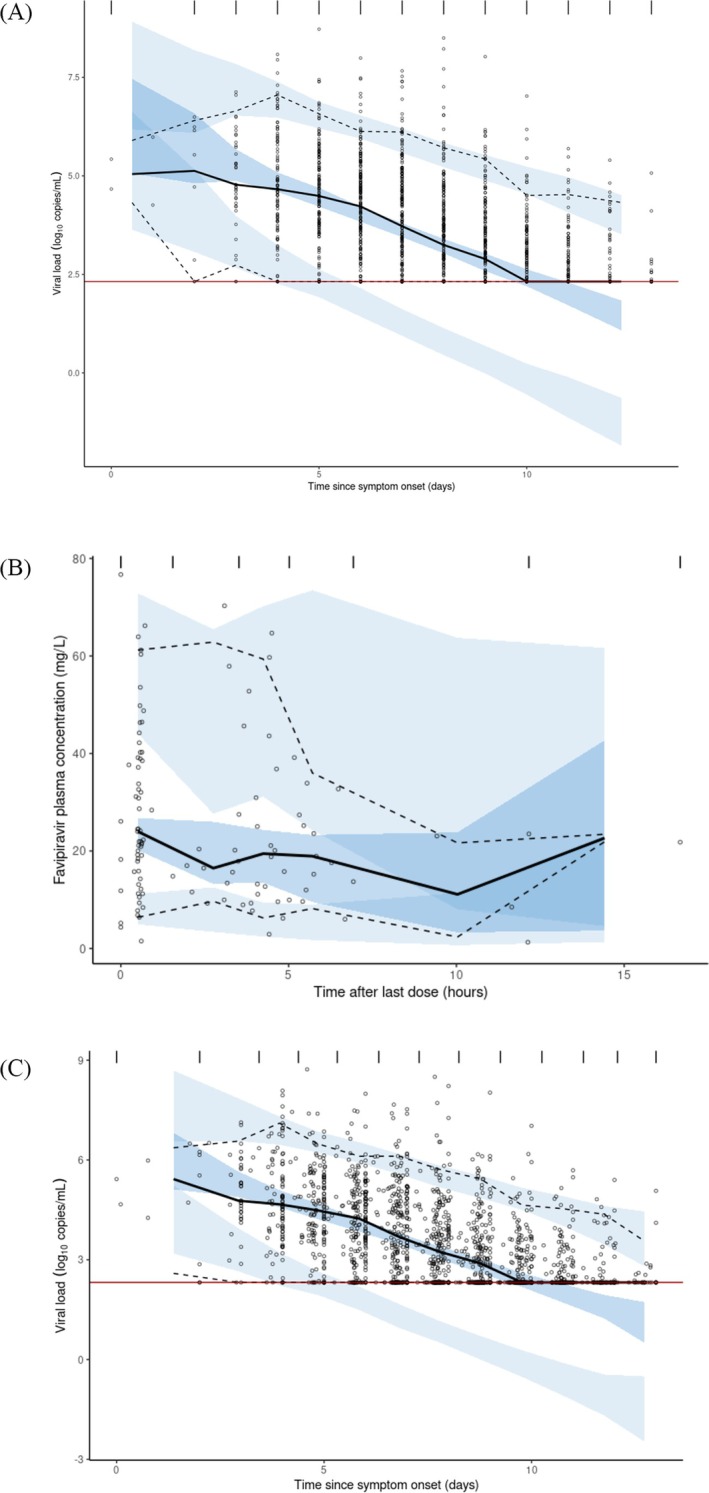
(A) Visual predictive check (VPC) for slope–intercept exponential decay viral dynamic model, (B) prediction‐corrected VPC for population pharmacokinetic model for favipiravir and (C) VPC for final population PK/PD model. Black circles are observations. The red line indicates the lower limit of quantification. The black line is the 50th percentile of the observed data. Broken black lines represent the 95th and 5th percentiles of the observed data. The shaded areas represent the 90% prediction interval of the 95th, 50th and 5th percentiles of the simulated data.

A total of 235 participants (>80% Caucasians, 115 females) with 1597 viral load samples were included. The median age and body weight were 39 years (range 19–68 years) and 74 kg (range 48–124 kg), respectively. Of the 235 participants, 59 (26 females) with a total of 59 trough and 56 peak favipiravir samples were included in the PK analysis. All participants tested positive for COVID‐19, and except for one asymptomatic participant, were within 7 days of symptom onset. Symptom onset was therefore assumed to be at enrolment for the asymptomatic participant. Plots of the raw viral load and favipiravir plasma concentration data are shown in Figures 2 and 3, respectively. GOF and VPC plots for all models are shown in Figures 4 and 5, respectively. Parameter estimates for all models are summarized in Table 1.

**TABLE 1:** Summary of model parameters and population mean estimates.

Parameter (unit)	Definition	Estimate (95% CI)	RSE (%)	Shrinkage (%)
Viral dynamic model				
*δ* (day^−1^)	Death rate of infected cells	0.934 (0.859–1.02)	63	‐
*V*_0_ (log_10_ copies/mL)	Viral load at symptom onset	6.48 (6.19–6.77)	2.29	‐
IIV *δ* (%)	Interindividual variability on *δ* presented as a coefficient of variation	13.2	‐	−2.73
IIV *V*_0_	Interindividual variability on *V*_0_ presented as standard deviation	1.73	‐	−3.88
Additive (log_10_ copies/mL)	Residual error	0.919	‐	‐
Population PK model for favipiravir				
*k*_*a*_ (h^−1^)^a^	Absorption rate constant	1.5	Fixed	‐
*CL*/*F* (L/h)	Apparent clearance	1.73 (1.43–2.1)	17.9	‐
*V*/*F* (L)	Apparent volume of distribution	22.1 (16.1–30.5)	5.27	‐
*tCL*/*F* (%)^a^	Time‐dependent percentage increase in favipiravir *CL*/*F* per day since study initiation	6.14	Fixed	‐
IIV *CL*/*F* (%)	Interindividual variability on *CL*/*F* presented as coefficient of variation	53.2	‐	8.25
LPV/r on *CL*/*F*	Factor associated with the effect of lopinavir–ritonavir on *CL*/*F*	2.22 (1.66–2.97)	18.9	‐
Additive (mg/L)	Residual error	0.0986	‐	‐
Proportional	Residual error	0.364	‐	‐
Final population PK/PD model				
*δ* (day^−1^)^b^	Death rate of infected cells	0.96 (0.883–1.04)	106	‐
*V*_0_ (log_10_ copies/mL)	Viral load at symptom onset	6.49 (6.19–6.78)	2.31	‐
IIV *δ* (%)	Interindividual variability on *δ* presented as a coefficient of variation	13	‐	−1.45
IIV *V*_0_	Interindividual variability on *V*_0_ presented as standard deviation	1.73	‐	−4.90
Additive (log_10_ copies/mL)	Residual error	0.918	‐	‐

Abbreviations: CI, confidence interval; LPV/r, lopinavir–ritonavir; PD, pharmacodynamic; PK, pharmacokinetic; RSE, relative standard error.

^a^ Parameters fixed to literature values.^24^

^b^ Bootstrap median (95% CI), %RSE: 0.96 (0.889–1.04), 97.9.

### 3.1 Viral dynamic model

After individually fitting the SI, rTCL, TCL and TCLE models to viral load data, the TCLE model with an incubation period of 4 days achieved the lowest BIC (TCLE [4‐day incubation period] = 5844 *vs*. SI = 5897, rTCL = 6275 and TCL [1‐day incubation period] = 5958). The median *δ* estimate across all models was 1.08 day^−1^ (range 0.68–2 day^−1^) (Table S1). All models showed satisfactory GOF plots. However, the VPC of the SI model achieved the best fit when the observed percentiles were compared to the prediction interval of the simulated data (Figure S1). Hence, the SI model was selected as the model that best characterized the viral load trajectory based on the biologically plausible *δ* estimate (0.934 day^−1^, 95% confidence interval [CI]: 0.859–1.02 day^−1^) and VPC for further covariate analysis. The effect of weight, age and sex on *δ* or baseline viral load was excluded from the final model following covariate assessment as described above.

### 3.2 Population PK model for favipiravir

A summary of the population PK model development steps is shown in Table S2. The structural model consisted of a one‐compartment model with first‐order absorption and elimination. After evaluation of IIV on *k*_*a*_, *CL*/*F* and *V*/*F*, only IIV on *CL*/*F* achieved the lowest OFV. Inclusion of a transit compartment and evaluation of a two‐compartment model increased the BIC by 26 and 22 units, respectively. *k*_*a*_ was poorly estimated (% residual standard error [RSE] = 91%) and therefore fixed to a literature‐derived value of 1.5 h^−1^.^24^ The estimated mean *CL*/*F* and *V*/*F* were 1.73 L/h (95% CI: 1.43–2.1 L/h) and 22.1 L (95% CI: 16.1–30.5 L), respectively. Of the covariates tested on *CL*/*F*, only co‐administration of lopinavir–ritonavir significantly increased the *CL*/*F* of favipiravir. About a twofold increase in *CL*/*F* was estimated, which consequently resulted in a lower predicted mean favipiravir plasma trough concentration in the combination arm (6.7 mg/L *vs*. monotherapy = 10.5 mg/L). The predicted secondary PK parameters are presented in Table S3. GOF plots were satisfactory. The prediction‐corrected VPC showed that the 5th, 50th and 95th percentiles of the observed data fell within the 90% prediction interval of the simulated data percentiles, indicating a good predictive performance of the final model.

### 3.3 Final population PK/PD model

The *δ* estimate in the joint model (0.96 day^−1^, 95% CI: 0.883–1.04 day^−1^) was similar to that of the viral dynamic model alone (0.934 day^−1^, 95% CI: 0.859–1.02 day^−1^). The bootstrap procedure also yielded a *δ* estimate of 0.96 day^−1^ (95% CI: 0.889–1.04 day^−1^) not different from those estimated from the original dataset. The inclusion of favipiravir and lopinavir–ritonavir drug effects as single agents and in combination did not improve model fit and were therefore excluded from the final model. GOF plots showed satisfactory agreement between the predicted and observed viral load trajectories. The VPC plot indicated acceptable agreement between the percentiles of the observed data and the 90% prediction interval of the simulated data percentiles. To further confirm the lack of antiviral effect on viral production rate, the favipiravir drug effect was tested on viral production rate in the TCLE model. This yielded an increase in OFV by ~25 units when compared with the TCLE with no drug effect.

### 3.4 Treatment effect on viral mutagenesis

A total of 203 whole‐genome sequence samples (137 participants) passed the quality control (i.e., >90% coverage with >10× mean read depth) with 57 participants having more than one time point (Table S4). Lineage analysis assigned between 65% and 70% to the delta variant (favipiravir/lopinavir–ritonavir [70.3%], favipiravir [68.7%], lopinavir–ritonavir [65.6%] and placebo [66.6%] arms) (Table S5).

**FIGURE 6:**
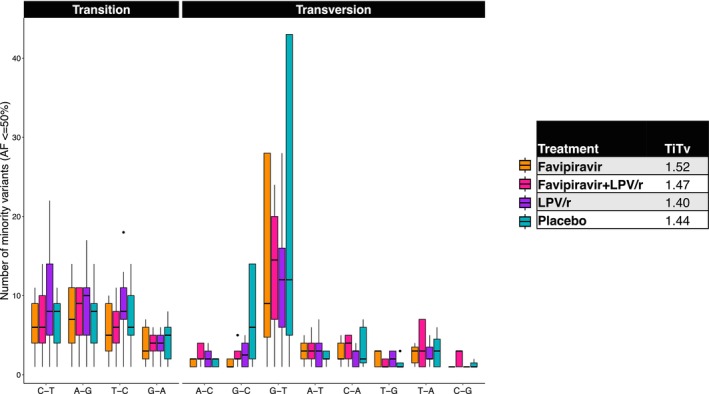
Total transition and transversion mutations over time in viral sequences. Box plots represent the total number of minority variants (5%–50% allele frequency [AF]) detected in samples collected from participants, divided by treatment group and type of nucleotide substitution. A total of 203 samples (including baseline) were analysed. LPV/r, lopinavir–ritonavir; TiTv, transition/transversion ratio.

**FIGURE 7:**
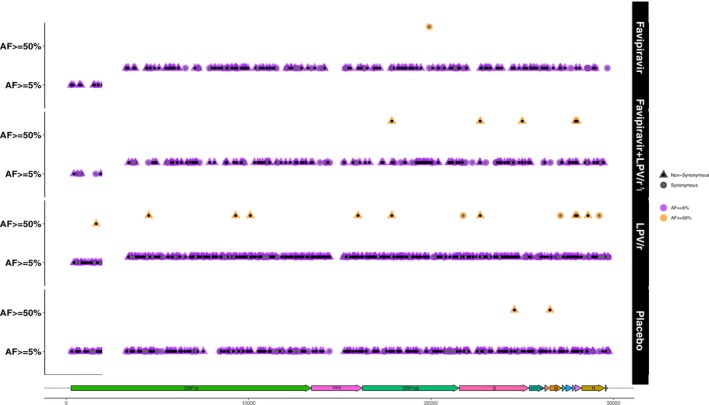
Post‐baseline whole‐genome mutational landscape highlighting in yellow above allele frequency [AF] >50% and in purple below AF ≤ 50% (between 5 to 50%) consensus mutations for favipiravir, favipiravir + lopinavir–ritonavir (LPV/r), LPV/r and placebo treatment arms. Circles with grey spots indicate synonymous mutations, whilst triangles with dark spots are non‐synonymous mutations.

We observed no increase in minority variants (allele frequency ≤50%) in the favipiravir and favipiravir/lopinavir–ritonavir arms. However, the number of transition mutations and the transition/transversion ratio were comparable across all arms (Figure 6). The impact of post‐baseline missense and stop codon mutations was classified as high in 50 out of the 57 participants (7, 12, 15 and 16 in the favipiravir, favipiravir/lopinavir–ritonavir, lopinavir–ritonavir and placebo arms, respectively). Most mutations were below the consensus level (allele frequency ≤50%) and non‐synonymous in all except the favipiravir monotherapy arm (Figure 7).

## 4 DISCUSSION

In this study, we developed a population PK/PD model in patients with COVID‐19 receiving early treatment with favipiravir alone or in combination with lopinavir–ritonavir to characterize the observed variability in favipiravir plasma concentrations and viral dynamics. This was a placebo‐controlled 2 × 2 factorial study that opened to recruitment in September 2020 despite not being a UK government priority trial. This shows that high‐quality clinical pharmacology studies can be quickly initiated during a pandemic.^10^ We found that baseline viral loads were not influenced by age or sex. Co‐administration of lopinavir–ritonavir was associated with increased clearance of favipiravir. Neither favipiravir nor lopinavir–ritonavir antiviral effect influenced the death rate of infected cells, and no clear mutagenic signature was observed following viral sequencing.

The SI model best described the observed viral dynamics when compared with the more complex models. The model was robust, having the same estimated *δ* (0.96 day^−1^) in the final joint model as the bootstrap of 200 runs. Also, this estimate is consistent with previous reports of 0.96^8^ and 0.93 day^−1^.^21^, ^31^ Both simple and complex models have been used to describe the viral dynamics of SARS‐CoV‐2 across varying patient populations.^20^, ^21^, ^22^, ^31^, ^32^, ^33^, ^34^ The viral load trajectory of the sample population and the identifiability of included model parameters support the model choice as appropriate. In our population, patients were recruited after symptom onset and therefore lacked viral loads prior to symptom onset to enable us to model the viral load trajectory earlier since time of infection. Hence, the SI model, which minimizes the inclusion of poorly identifiable parameters, was sufficient to characterize the observed post‐symptomatic viral load trajectory.

Of note, published complex models on SARS‐CoV‐2 viral dynamics have fixed most of the model parameters to ensure parameter identifiability. Nonetheless, such model complexities are inevitable in certain instances. For example, where viral loads are obtained prior to symptom onset,^35^ the SI model may not provide a good model fit. Additionally, parsimonious models will benefit from extensions to capture immune responses, which are important within‐host processes influencing viral dynamics.^33^, ^34^

We found no significant influence of age and sex on the baseline viral loads. Inconsistent results have been reported from previous studies on the association between SARS‐CoV‐2 viral loads and age or sex.^36^, ^37^, ^38^, ^39^, ^40^, ^41^ Whilst our results are consistent with a previous report from Challenger et al.,^34^ another possible explanation is that we studied mainly younger patients during their first infection where age and sex variations, which seem to be mediated by antibody levels, are not so apparent.^8^

Regarding favipiravir pharmacokinetics, a one‐compartment model with first‐order absorption and elimination best described the observed plasma concentrations. Lower *CL*/*F* was observed in our population compared to earlier reports for COVID‐19^16^ and severe influenza.^24^ Nonetheless, our estimates were of good precision (%RSE < 30% for *CL*/*F* and *V*/*F*), which indicates that the model well characterized the observed favipiravir plasma concentrations, and with lower shrinkage on *CL*/*F* also depicting reliable external predictions. Differences in favipiravir pharmacokinetics have been reported between Japanese and US healthy adult subjects following either a single or multiple oral dosage regimen. The study attributed the differences to be possibly due to differences in restoration of the aldehyde oxidase activity between Japanese and US subjects.^42^ Also, studies in healthy Egyptian adults have reported similar favipiravir pharmacokinetics to those observed in US subjects but different from Japanese subjects.^43^, ^44^ Thus, the difference in *CL*/*F* of our population, which was dominated by Caucasians (>80%), compared to other COVID‐19 populations could potentially be due to ethnic differences and adds to the growing knowledge of the impact of ethnic differences on favipiravir pharmacokinetics.

Lopinavir–ritonavir was found to significantly cause an estimated twofold increase in favipiravir *CL*/*F*, resulting in ~2‐fold decrease in the predicted favipiravir trough concentrations in the combination arm compared with monotherapy. This prediction agrees with the observed ~2‐fold decreased favipiravir trough concentrations in the combination arm compared to monotherapy in our previous report.^12^ The possibility of a potential drug–drug interaction may account for this observation, although no known drug–drug interaction between favipiravir and lopinavir–ritonavir has yet been reported.^45^ Hence, further investigations may be required to establish any potential clinical significance.

The inclusion of a stimulatory drug effect on *δ* in the final PK/PD model did not well characterize the observed data. This implies that favipiravir lacks antiviral effect either as monotherapy or in combination with lopinavir–ritonavir against SARS‐CoV‐2 with the current dosing regimen. A similar dosing regimen for favipiravir was also investigated in the PLATCOV (open‐label, randomized, controlled, adaptive platform) trial and showed a lack of antiviral activity in early treatment for COVID‐19.^15^ Also, although studies using hamster models have reported favipiravir efficacy against SARS‐CoV‐2 via its mutagenic action,^46^, ^47^ in our study, the frequency of transition mutations in both the favipiravir monotherapy and favipiravir/lopinavir–ritonavir arms was comparable to the placebo and lopinavir–ritonavir monotherapy arms. One possible explanation might be that the dose of antiviral given was insufficient to induce mutagenesis.^48^ Therefore, further investigations to explore the optimal dosing regimen for favipiravir in COVID‐19 are warranted.

The present study has some limitations. The sample size for the PK analysis was small and dominated by Caucasians. In addition, the stepwise covariate modelling approach utilized in the current study may have yielded limited outcomes compared to alternative machine learning‐based covariate modelling approaches.^49^ Therefore, our findings may not entirely extend to other populations. Also, we used plasma favipiravir concentrations rather than intracellular concentrations of favipiravir active metabolite. Although intracellular concentrations are not representative of unbound effective concentration, further studies could explore the relationship between favipiravir plasma and intracellular concentrations to give a better understanding of the complex pharmacokinetics associated with favipiravir. Lastly, we did not test the influence of host immune response on the control of viral load, and thus, future studies with sufficient immunological data may explore this further.

In conclusion, the present study has applied a population PK/PD modelling approach and whole‐genome sequencing to give a better understanding of the lack of clinical benefit following the repurposed use of favipiravir alone or in combination with lopinavir–ritonavir for early treatment in COVID‐19. The results here are useful to inform the design of future studies investigating favipiravir alone or in combination with other agents for COVID‐19.

## AUTHOR CONTRIBUTIONS

A.A.A., J.F.S. and L.B. wrote the manuscript; J.F.S., D.M.L., J.B., A.A.A. and L.B. designed the research; D.M.L. and L.K.B. performed the research; A.A.A., J.F.S., J.B., L.B. and J.A.G. analysed the data. All authors were involved in the preparation of the final manuscript.

## CONFLICT OF INTEREST STATEMENT

The authors declare no conflict of interest.

## Supporting information


**Table S1.**Summary of model parameters and population mean estimates for viral dynamic model assessment.

**Table S2.**Summary of population pharmacokinetic model development steps for favipiravir.

**Table S3.**Predicted secondary pharmacokinetic parameters following a single dose of 400 mg favipiravir.

**Table S4.**Summary of sequenced samples at each time point.

**Table S5.**Summary of lineage assignment of baseline samples.

**Figure S1.** Goodness‐of‐fit and visual predictive check plots for viral dynamic model assessment.

**Appendix S1:** FLARE investigators list.

## Data Availability

Data and R scripts for reproducing the analysis are available at https://github.com/ucl-pharmacometrics/FLARE_PKPD_analysis_BJCP_final. Study whole‐genome sequencing data are available at NIH BioProject ID PRJNA1132166.

## References

[1] Lamontagne F , Agarwal A , Rochwerg B , et al. A living WHO guideline on drugs for covid‐19. BMJ. 2020;370:m3379. doi:10.1136/bmj.m3379 32887691

[2] Najjar‐Debbiny R , Gronich N , Weber G , et al. Effectiveness of Paxlovid in reducing severe coronavirus disease 2019 and mortality in high‐risk patients. Clin Infect Dis. 2023;76(3):e342‐e349. doi:10.1093/cid/ciac443 35653428 PMC9214014

[3] Du Z , Wang L , Bai Y , et al. A retrospective cohort study of Paxlovid efficacy depending on treatment time in hospitalized COVID‐19 patients. Elife. 2024;13:13. doi:10.7554/eLife.89801 PMC11078542 38622989

[4] Schilling WHK , Jittamala P , Watson JA , et al. Antiviral efficacy of molnupiravir versus ritonavir‐boosted nirmatrelvir in patients with early symptomatic COVID‐19 (PLATCOV): an open‐label, phase 2, randomised, controlled, adaptive trial. Lancet Infect Dis. 2024;24(1):36‐45. doi:10.1016/S1473-3099(23)00493-0 37778363 PMC7615401

[5] Jayk Bernal A , Gomes da Silva MM , Musungaie DB , et al. Molnupiravir for oral treatment of Covid‐19 in nonhospitalized patients. N Engl J Med. 2022;386(6):509‐520. doi:10.1056/NEJMoa2116044 34914868 PMC8693688

[6] Wang L , Berger NA , Davis PB , Kaelber DC , Volkow ND , Xu R . COVID‐19 rebound after Paxlovid and molnupiravir during January–June 2022. medRxiv. 2022;2022.06.21.22276724. doi:10.1101/2022.06.21.22276724

[7] Harrington PR , Cong J , Troy SB , et al. Evaluation of SARS‐CoV‐2 RNA rebound after nirmatrelvir/ritonavir treatment in randomized, double‐blind, placebo‐controlled trials—United States and international sites, 2021–2022. MMWR Morb Mortal Wkly Rep. 2023;72(51):1365‐1370. doi:10.15585/mmwr.mm7251a2 38127674 PMC10754264

[8] Standing JF , Buggiotti L , Guerra‐Assuncao JA , et al. Randomized controlled trial of molnupiravir SARS‐CoV‐2 viral and antibody response in at‐risk adult outpatients. Nat Commun. 2024;15(1):1652. doi:10.1038/s41467-024-45641-0 38396069 PMC10891158

[9] Martinez MA . Efficacy of repurposed antiviral drugs: lessons from COVID‐19. Drug Discov Today. 2022;27(7):1954‐1960. doi:10.1016/j.drudis.2022.02.012 35192924 PMC8857759

[10] Standing JF , Agyeman AA . Learning and confirming in publicly funded antiviral trials. Lancet Infect Dis. 2023;23(2):132‐133. doi:10.1016/S1473-3099(22)00665-X 36272434 PMC9581520

[11] Arshad U , Pertinez H , Box H , et al. Prioritization of anti‐SARS‐Cov‐2 drug repurposing opportunities based on plasma and target site concentrations derived from their established human pharmacokinetics. Clin Pharmacol Ther. 2020;108(4):775‐790. doi:10.1002/cpt.1909 32438446 PMC7280633

[12] Lowe DM , Brown L‐AK , Chowdhury K , et al. Favipiravir, lopinavir‐ritonavir, or combination therapy (FLARE): a randomised, double‐blind, 2 × 2 factorial placebo‐controlled trial of early antiviral therapy in COVID‐19. PLoS Med. 2022;19(10):e1004120. doi:10.1371/journal.pmed.1004120 36260627 PMC9629589

[13] Ison MG , Scheetz MH . Understanding the pharmacokinetics of favipiravir: implications for treatment of influenza and COVID‐19. EBioMedicine. 2021;63:103204. doi:10.1016/j.ebiom.2020.103204 33418497 PMC7785424

[14] Siripongboonsitti T , Muadchimkaew M , Tawinprai K , et al. Favipiravir treatment in non‐severe COVID‐19: promising results from multicenter propensity score‐matched study (FAVICOV). Sci Rep. 2023;13(1):14884. doi:10.1038/s41598-023-42195-x 37689754 PMC10492810

[15] Luvira V , Schilling WHK , Jittamala P , et al. Clinical antiviral efficacy of favipiravir in early COVID‐19 (PLATCOV): an open‐label, randomised, controlled, adaptive platform trial. BMC Infect Dis. 2024;24(1):89. doi:10.1186/s12879-023-08835-3 38225598 PMC10789040

[16] Irie K , Nakagawa A , Fujita H , et al. Population pharmacokinetics of favipiravir in patients with COVID‐19. CPT Pharmacometrics Syst Pharmacol. 2021;10(10):1161‐1170. doi:10.1002/psp4.12685 34292670 PMC8420316

[17] Gülhan R , Eryüksel E , Gülçebi İdriz Oğlu M , et al. Pharmacokinetic characterization of favipiravir in patients with COVID‐19. Br J Clin Pharmacol. 2022;88(7):3516‐3522. doi:10.1111/bcp.15227 35014080

[18] Laouénan C , Guedj J , Mentré F . Clinical trial simulation to evaluate power to compare the antiviral effectiveness of two hepatitis C protease inhibitors using nonlinear mixed effect models: a viral kinetic approach. BMC Med Res Methodol. 2013;13:60. doi:10.1186/1471-2288-13-60 23617810 PMC3651343

[19] Dodds MG , Krishna R , Goncalves A , Rayner CR . Model‐informed drug repurposing: viral kinetic modelling to prioritize rational drug combinations for COVID‐19. Br J Clin Pharmacol. 2021;87(9):3439‐3450. doi:10.1111/bcp.14486 32693436 PMC8451752

[20] Gonçalves A , Bertrand J , Ke R , et al. Timing of antiviral treatment initiation is critical to reduce SARS‐CoV‐2 viral load. CPT Pharmacometrics Syst Pharmacol. 2020;9(9):509‐514. doi:10.1002/psp4.12543 32558354 PMC7323384

[21] Kim KS , Ejima K , Iwanami S , et al. A quantitative model used to compare within‐host SARS‐CoV‐2, MERS‐CoV, and SARS‐CoV dynamics provides insights into the pathogenesis and treatment of SARS‐CoV‐2. PLoS Biol. 2021;19(3):e3001128. doi:10.1371/journal.pbio.3001128 33750978 PMC7984623

[22] Agyeman AA , You T , Chan PLS , et al. Comparative assessment of viral dynamic models for SARS‐CoV‐2 for pharmacodynamic assessment in early treatment trials. Br J Clin Pharmacol. 2022;88(12):5428‐5433. doi:10.1111/bcp.15518 36040430 PMC9538685

[23] Fiddler M . Censoring in nlmixr. 2023. https://nlmixr2.org/articles/censoring.html

[24] Wang Y , Zhong W , Salam A , et al. Phase 2a, open‐label, dose‐escalating, multi‐center pharmacokinetic study of favipiravir (T‐705) in combination with oseltamivir in patients with severe influenza. EBioMedicine. 2020;62:103125. doi:10.1016/j.ebiom.2020.103125 33232871 PMC7689521

[25] Anderson BJ , Holford NH . Mechanistic basis of using body size and maturation to predict clearance in humans. Drug Metab Pharmacokinet. 2009;24(1):25‐36. 19252334 10.2133/dmpk.24.25

[26] Alvarez JC , Moine P , Davido B , et al. Population pharmacokinetics of lopinavir/ritonavir in Covid‐19 patients. Eur J Clin Pharmacol. 2021;77(3):389‐397. doi:10.1007/s00228-020-03020-w 33048175 PMC7552959

[27] Dickinson L , Boffito M , Back D , et al. Sequential population pharmacokinetic modeling of lopinavir and ritonavir in healthy volunteers and assessment of different dosing strategies. Antimicrob Agents Chemother. 2011;55(6):2775‐2782. doi:10.1128/AAC.00887-10 21422211 PMC3101471

[28] Patel H , Monzón S , Varona S , et al. nf‐core/viralrecon: nf‐core/viralrecon v2.6.0—Rhodium Raccoon. 2023. 10.5281/zenodo.7764938

[29] Garrison E , Marth G . Haplotype‐based variant detection from short‐read sequencing. 2012. https://arxiv.org/pdf/1207.3907

[30] Alexander SPH , Kelly E , Mathie A , et al. The Concise Guide to PHARMACOLOGY 2021/22: introduction and other protein targets. Br J Pharmacol. 2021;178(S1):S1‐S26. 34529830 10.1111/bph.15537 PMC9513948

[31] Néant N , Lingas G , Le Hingrat Q , et al. Modeling SARS‐CoV‐2 viral kinetics and association with mortality in hospitalized patients from the French COVID cohort. Proc Natl Acad Sci USA. 2021;118(8):e2017962118. doi:10.1073/pnas.2017962118 33536313 PMC7929555

[32] Cao Y , Gao W , Caro L , Stone JA . Immune‐viral dynamics modeling for SARS‐CoV‐2 drug development. Clin Transl Sci. 2021;14(6):2348‐2359. doi:10.1111/cts.13099 34121337 PMC8444857

[33] Goyal A , Cardozo‐Ojeda EF , Schiffer JT . Potency and timing of antiviral therapy as determinants of duration of SARS‐CoV‐2 shedding and intensity of inflammatory response. Sci Adv. 2020;6(47):eabc7112. doi:10.1126/sciadv.abc7112 33097472 PMC7679107

[34] Challenger JD , Foo CY , Wu Y , et al. Modelling upper respiratory viral load dynamics of SARS‐CoV‐2. BMC Med. 2022;20(1):25. doi:10.1186/s12916-021-02220-0 35022051 PMC8755404

[35] Kissler SM , Fauver JR , Mack C , et al. Viral dynamics of acute SARS‐CoV‐2 infection and applications to diagnostic and public health strategies. PLoS Biol. 2021;19(7):e3001333. doi:10.1371/journal.pbio.3001333 34252080 PMC8297933

[36] Jacot D , Greub G , Jaton K , Opota O . Viral load of SARS‐CoV‐2 across patients and compared to other respiratory viruses. Microbes Infect. 2020;22(10):617‐621. doi:10.1016/j.micinf.2020.08.004 32911086 PMC7476607

[37] Mahallawi WH , Alsamiri AD , Dabbour AF , Alsaeedi H , Al‐Zalabani AH . Association of viral load in SARS‐CoV‐2 patients with age and gender. Front Med (Lausanne). 2021;8:608215. doi:10.3389/fmed.2021.608215 33585523 PMC7873591

[38] To KK , Tsang OT , Leung WS , et al. Temporal profiles of viral load in posterior oropharyngeal saliva samples and serum antibody responses during infection by SARS‐CoV‐2: an observational cohort study. Lancet Infect Dis. 2020;20(5):565‐574. doi:10.1016/S1473-3099(20)30196-1 32213337 PMC7158907

[39] Heald‐Sargent T , Muller WJ , Zheng X , Rippe J , Patel AB , Kociolek LK . Age‐related differences in nasopharyngeal severe acute respiratory syndrome coronavirus 2 (SARS‐CoV‐2) levels in patients with mild to moderate coronavirus disease 2019 (COVID‐19). JAMA Pediatr. 2020;174(9):902‐903. doi:10.1001/jamapediatrics.2020.3651 32745201 PMC7393583

[40] Kleiboeker S , Cowden S , Grantham J , et al. SARS‐CoV‐2 viral load assessment in respiratory samples. J Clin Virol. 2020;129:104439. doi:10.1016/j.jcv.2020.104439 32674034 PMC7235577

[41] Takahashi T , Ellingson MK , Wong P , et al. Sex differences in immune responses that underlie COVID‐19 disease outcomes. Nature. 2020;588(7837):315‐320. doi:10.1038/s41586-020-2700-3 32846427 PMC7725931

[42] PMDA . Report on the deliberation results. Evaluation and Licensing Division, Pharmaceutical and Food Safety Bureau Ministry of Health, Labour and Welfare. 2014. https://www.pmda.go.jp/files/000210319.pdf

[43] Bendas ER , Rezk MR , Badr KA . Does the ethnic difference affect the pharmacokinetics of favipiravir? A pharmacokinetic study in healthy Egyptian volunteers and development of level C in‐vitro in‐vivo correlation. Drug Res (Stuttg). 2023;73(6):349‐354. doi:10.1055/a-2061-7074 37094796

[44] Salem A , Gouda A , Marzouk H , Rezk M , Abdel‐Megied A . A population pharmacokinetic modeling approach to evaluate the current dosing recommendations of favipiravir in Egyptian subjects. Int J Infect Dis. 2023;130:S94. doi:10.1016/j.ijid.2023.04.234

[45] The University of Liverpool . HIV drug interactions. 2024. https://www.hiv-druginteractions.org/

[46] Driouich JS , Cochin M , Lingas G , et al. Favipiravir antiviral efficacy against SARS‐CoV‐2 in a hamster model. Nat Commun. 2021;12(1):1735. doi:10.1038/s41467-021-21992-w 33741945 PMC7979801

[47] Kaptein SJF , Jacobs S , Langendries L , et al. Favipiravir at high doses has potent antiviral activity in SARS‐CoV‐2‐infected hamsters, whereas hydroxychloroquine lacks activity. Proc Natl Acad Sci USA. 2020;117(43):26955‐26965. doi:10.1073/pnas.2014441117 33037151 PMC7604414

[48] Illingworth CJR , Guerra‐Assuncao JA , Gregg S , et al. Genetic consequences of effective and suboptimal dosing with mutagenic drugs in a hamster model of SARS‐CoV‐2 infection. Virus Evol. 2024;10(1):veae001. doi:10.1093/ve/veae001 38486802 PMC10939363

[49] Karlsen M , Khier S , Fabre D , et al. Covariate model selection approaches for population pharmacokinetics: a systematic review of existing methods, from SCM to AI. CPT Pharmacomet Syst Pharmacol. 2025;14(4):621‐639. 10.1002/psp4.13306 PMC12001260 39831409

