# Supplementary material for: Population pharmacokinetic/pharmacodynamic modelling to evaluate favipiravir in combination with lopinavir–ritonavir in patients with COVID‐19
Source: Br J Clin Pharmacol. 2026 Mar 23;92(7):2390–402. doi: 10.1002/bcp.70507 (PMC13304270; doi:10.1002/bcp.70507)
Supplement: Supplementary file 1 — Table S1.Summary of model parameters and population mean estimates for viral dynamic model assessment. [file BCP-92-2390-s007.pdf]

Table S1. Summary of model parameters and population mean estimates for viral dynamic model assessment.

**Model Parameters**	**Fixed effects – Population mean estimate (95% CI) (%RSE)**				
**Parameter (Symbol, Unit)**	**SI**	**rTCL**	**TCL**	**TCLE**	**TCLE***
Death rate of infected cells (δ, day^{-1})	0.934 (0.859-1.02) (63)	0.684 (0.614-0.762) (14.5)	1.23 (1.2-1.27) (7.64)	2 (1.16-3.45) 40.4	3.45 (2.38-5.01) 15.3
Viral load at symptom onset (V_0, $\log_{10}$ copies/mL)	6.48 (6.19-6.77) (2.29)	5.67 (5.53-5.81) (1.26)	-	-	-
Rate constant for virus infection (β, ((copies/ml) $^{-1}$.day^{-1}))	-	0.0322 (0.00283-0.366) (36.1)	3.33×10^{-5} (1.99×10^{-5}-5.57×10^{-5}) (2.55)	1.36×10^{-5} (4.46×10^{-5}-4.15×10^{-5}) (5.08)	1.71×10^{-5} (9.07×10^{-6}-3.22×10^{-5}) (2.95)
Viral production rate (ρ, copies/cell.day^{-1})	-	-	39.5 (3.12-499) (35.2)	312 (56.2-1.74×10^{3}) (15.2)	133 (60.5-291) (8.2)
Viral clearance rate (c, day^{-1})	-	-	1.59 (1.42-1.79) (12.8)	3.04 (1.45-6.35) (33.8)	2.55 (1.72-3.78) (21.5)
Maximum rate constant for viral replication (γ, day^{-1})	-	1 (fixed)	-	-	-
Reproductivity rate of infected cells (k, day^{-1})	-	-	-	1.01 (0.873-1.16) 969	0.969 (0.823-1.14) 263
beta Factor associated with effect of favipiravir on ρ	-	-	-	-	1.220 (0.552–2.296) (307)
Additive residual error ($\log_{10}$ copies/mL)	0.919	0.949	0.956	0.959	0.974
**Random effects**	**Coefficient of variation (%) (%Shrinkage)**				
IIV δ	13.2 (-2.73)	14.7 (47.5)	23 (37)	5.44 (90.2)	90.5 (51.8)
IIV V_0	1.73 (-3.88)	2.24 (-14.4)	-	-	-
IIV β	-	26.6 (98.2)	50.2 (71.1)	5.22 (94.5)	14.4 (86.1)
IIV ρ	-	-	5.01 (97.6)	3.76 (96.5)	4.17 (97.3)
IIV c	-	-	80.5 (20.6)	72.2 (48.7)	8.6 (93.7)
IIV γ	-	-	-	-	-
IIV k	-	-	-	48.7 (22.3)	47.2 (19.2)

SI, slope-intercept exponential decay model; rTCL, reduced target cell limited model; TCL, target cell limited model; TCLE, TCL with eclipse phase; CI, confidence interval; RSE, residual standard error; IIV, inter-individual variability.

-, not applicable.

*Parameter estimates for TCLE model accounting for favipiravir drug effect tested on viral production rate.
