# Supplementary material for: Population pharmacokinetic/pharmacodynamic modelling to evaluate favipiravir in combination with lopinavir–ritonavir in patients with COVID‐19
Source: Br J Clin Pharmacol. 2026 Mar 23;92(7):2390–402. doi: 10.1002/bcp.70507 (PMC13304270; doi:10.1002/bcp.70507)
Supplement: Supplementary file 2 — Table S2.Summary of population pharmacokinetic model development steps for favipiravir. [file BCP-92-2390-s001.pdf]

Table S2. Summary of population pharmacokinetic model development steps for favipiravir

Model	Model compared to	Description	OFV	Change in OFV	AIC	Change in AIC	BIC	Change in BIC
1	-	1-cmt combined additive and proportional residual error + ALLCL/F+ALLV/F + tCL/F + IIVCL/F	678.391	-	901.747	-	918.216	-
2	-	1-cmt combined additive and proportional residual error + ALLCL/F+ALLV/F + tCL/F + IIVka	774.274	-	997.63	-	1014.1	-
3	-	1-cmt combined additive and proportional residual error + ALLCL/F+ALLV/F + tCL/F + IIVV/F	817.148	-	1040.504	-	1056.974	-
4	1	1-cmt combined additive and proportional residual error + ALLCL/F+ALLV/F+ tCL/F + IIVCL/F+IIVka	718.565	40.175	943.921	42.175	963.136	44.92
5	1	1-cmt combined additive and proportional residual error + ALLCL/F+ALLV/F + tCL/F + IIVCL/F+IIVV/F	694.465	16.075	919.821	18.075	939.036	20.82
6	1	1-cmt combined additive and proportional residual error + ALLCL/F+ALLV/F + tCL/F + IIVCL/F+IIVV/F+IIVka	699.958	21.567	927.314	25.567	949.274	31.057
7	1	1-cmt combined additive and proportional residual error + ALLCL/F+ALLV/F + tCL/F + IIVCL/F+ tcmt	695.078	16.688	922.434	20.688	944.394	26.177
8	1	2-cmt combined additive and proportional residual error + ALLCL/F+ALLV/F + tCL/F + IIVCL/F	681.085	2.695	912.441	10.695	939.891	21.674
9 (Base)	1	1-cmt combined additive and proportional residual error + ALLCL/F+ALLV/F + tCL/F + IIVCL/F + fixka	681.673	3.282	903.029	1.282	916.754	-1.462
**10 (Final)**	**9**	**Base with LPVr_cat on CL/F**	**657.222**	**-24.451**	**880.578**	**-22.451**	**897.048**	**-19.706**
11	10	Base with LPVr_cat and age on CL/F	654.719	-2.503	880.075	-0.503	899.29	2.242
12	10	Base with LPVr_cat and bilirubin on CL/F	657.318	0.096	882.674	2.096	901.889	4.841
13	10	Base with LPVr_cat and alkaline phosphatase on CL/F	657.178	-0.044	882.534	1.956	901.748	4.701

14	10	Base with LPVr_cat and albumin on CL/F	657.213	-0.009	882.569	1.991	901.783	4.735
15	10	Base with LPVr_cat and uric acid on CL/F	655.677	-1.546	881.032	0.454	900.247	3.199
16	10	Base with LPVr_cat and serum creatinine on CL/F	650.731	-6.492	876.087	-4.492	895.301	-1.747
17	16	Base with LPVr_cat and serum creatinine and alanine transaminase on CL/F	648.049	-2.682	875.405	-0.682	897.364	2.063
18	16	Base with LPVr_cat and serum creatinine and aspartate transaminase on CL/F	647.851	-2.88	875.207	-0.88	897.166	1.865
19	16	Back elimination of LPVr_cat from full Model 16	677.037	26.306	900.393	24.306	916.862	21.561
20	16	Back elimination of serum creatinine from full Model 16	657.2	6.47	880.556	4.47	897.026	1.725

ka, absorption rate constant; CL/F, apparent clearance; V/F, apparent volume of distribution; tCL/F, time dependent percentage increase in favipiravir CL/F per day since study initiation; IIV, interindividual variability; cmt, compartment; tcmt, transit compartment; ALL, allometric exponent; LPVr_cat, covariate effect of lopinavir-ritonavir on CL/F; Base, base model; OFV, objective function value; AIC, Akaike information criterion; BIC, Bayesian information criterion.
