# Supplementary material for: Population pharmacokinetic/pharmacodynamic modelling to evaluate favipiravir in combination with lopinavir–ritonavir in patients with COVID‐19
Source: Br J Clin Pharmacol. 2026 Mar 23;92(7):2390–402. doi: 10.1002/bcp.70507 (PMC13304270; doi:10.1002/bcp.70507)
Supplement: Supplementary file 3 — Table S3.Predicted secondary pharmacokinetic parameters following a single dose of 400 mg favipiravir. [file BCP-92-2390-s003.pdf]

Table S3. Predicted secondary pharmacokinetic parameters following a single dose of 400 mg favipiravir

**Parameter (unit)**	**Favipiravir**	**Favipiravir + lopinavir-ritonavir**
C_{max} (mg/L)	13.02	11.66
T_{max} (h)	2.2	1.7
C_{trough} (mg/L)	10.5	6.7
$AUC_{0\text{-}6h}$ (mg×h/L)	67.24	54.83

C_{max}, maximum plasma concentration; T_{max}, time to reach C_{max}; C_{trough}, minimum plasma concentration and $AUC_{0\text{-}6h}$, area under the concentration-time curve up to 6 hours.
