# Supplementary material for: Population pharmacokinetic/pharmacodynamic modelling to evaluate favipiravir in combination with lopinavir–ritonavir in patients with COVID‐19
Source: Br J Clin Pharmacol. 2026 Mar 23;92(7):2390–402. doi: 10.1002/bcp.70507 (PMC13304270; doi:10.1002/bcp.70507)
Supplement: Supplementary file 4 — Table S4.Summary of sequenced samples at each time point. [file BCP-92-2390-s002.pdf]

Table S4. Summary of sequenced samples at each time point[a]

Treatment	Number of days from baseline							Total samples	Total participants
	**0**	**1**	**2**	**3**	**4**	**5**	**6**		
Favipiravir	27	4	4	2	6	2		45	32
Favipiravir + LPV/r	33		3	6	8		1	51	37
LPV/r	31	3	3	5	6	2		50	32
Placebo	36	1	5	3	10	2		57	36
								**203**	**137**

[a]All baseline samples were taken pretreatment.

LPV/r, lopinavir-ritonavir.
