# Supplementary material for: Population pharmacokinetic/pharmacodynamic modelling to evaluate favipiravir in combination with lopinavir–ritonavir in patients with COVID‐19
Source: Br J Clin Pharmacol. 2026 Mar 23;92(7):2390–402. doi: 10.1002/bcp.70507 (PMC13304270; doi:10.1002/bcp.70507)
Supplement: Supplementary file 5 — Table S5.Summary of lineage assignment of baseline samples. [file BCP-92-2390-s005.pdf]

Table S5. Summary of lineage assignment of baseline samples

Treatment	Pangolin	Nextclade	Number of participants
Favipiravir	AY.10	21I (Delta)	1
Favipiravir	AY.120	21J (Delta)	1
Favipiravir	AY.3	21J (Delta)	1
Favipiravir	AY.4	21J (Delta)	10
Favipiravir	AY.4.2	21J (Delta)	1
Favipiravir	AY.46.5	21J (Delta)	1
Favipiravir	AY.5	21J (Delta)	4
Favipiravir	AY.9	21I (Delta)	1
Favipiravir	B.1	20A	1
Favipiravir	B.1.1.37	20B	1
Favipiravir	B.1.1.7	20I (Alpha; V1)	6
Favipiravir	B.1.258	20A	1
Favipiravir	B.1.408	20A	1
Favipiravir	B.1.617.2	21A (Delta)	1
Favipiravir	B.1.617.2	21J (Delta)	1
Favipiravir+LPV/r	AY.120	21J (Delta)	1
Favipiravir+LPV/r	AY.4	21J (Delta)	18
Favipiravir+LPV/r	AY.4.2	21J (Delta)	2
Favipiravir+LPV/r	AY.5	21J (Delta)	2
Favipiravir+LPV/r	AY.6	21J (Delta)	1
Favipiravir+LPV/r	AY.9	21I (Delta)	1
Favipiravir+LPV/r	B.1.1.7	20I (Alpha; V1)	9
Favipiravir+LPV/r	B.1.177	20E (EU1)	2
Favipiravir+LPV/r	B.1.617.2	21A (Delta)	1
LPV/r	AY.120	21J (Delta)	1
LPV/r	AY.4	21J (Delta)	15
LPV/r	AY.4.2	21J (Delta)	1
LPV/r	AY.5	21J (Delta)	2
LPV/r	AY.7	21J (Delta)	1
LPV/r	AY.9	21I (Delta)	1
LPV/r	B.1.1.7	20I (Alpha; V1)	9
LPV/r	B.1.258	20A	1
LPV/r	B.1.36.34	20A	1
Placebo	AY.27	21I (Delta)	1
Placebo	AY.4	21J (Delta)	11
Placebo	AY.4.1	21J (Delta)	1

Placebo	AY.43	21J (Delta)	2
Placebo	AY.5	21J (Delta)	4
Placebo	AY.6	21J (Delta)	1
Placebo	AY.90	21J (Delta)	1
Placebo	AY.98	21J (Delta)	2
Placebo	B.1.1.37	20B	1
Placebo	B.1.1.7	20I (Alpha; V1)	9
Placebo	B.1.177.33	20E (EU1)	1
Placebo	B.1.398	20A	1
Placebo	B.1.617.2	21A (Delta)	1

LPV/r, lopinavir-ritonavir.
