# Supplementary material for: Population pharmacokinetic/pharmacodynamic modelling to evaluate favipiravir in combination with lopinavir–ritonavir in patients with COVID‐19
Source: Br J Clin Pharmacol. 2026 Mar 23;92(7):2390–402. doi: 10.1002/bcp.70507 (PMC13304270; doi:10.1002/bcp.70507)
Supplement: Supplementary file 6 — Figure S1. Goodness‐of‐fit and visual predictive check plots for viral dynamic model assessment. [file BCP-92-2390-s004.pdf]

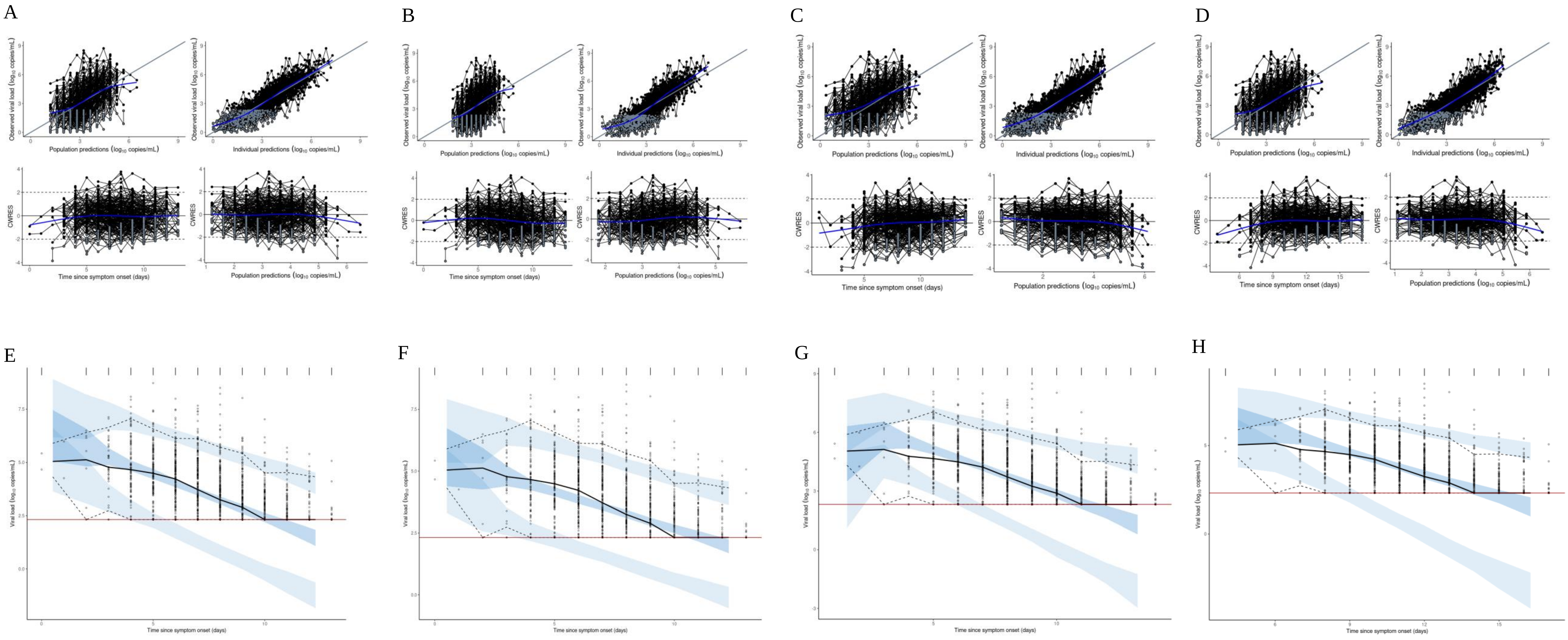

Figure S1. Goodness-of-fit (A-D) and visual predictive check (E-H) plots for slope-intercept exponential decay (A and E), reduced target cell limited (B and F), target cell limited (C and G) and target cell limited with eclipse phase (D and H) models.
