# Supplementary material for: Population pharmacokinetic/pharmacodynamic modelling to evaluate favipiravir in combination with lopinavir–ritonavir in patients with COVID‐19
Source: Br J Clin Pharmacol. 2026 Mar 23;92(7):2390–402. doi: 10.1002/bcp.70507 (PMC13304270; doi:10.1002/bcp.70507)
Supplement: Supplementary file 7 — Appendix S1: FLARE investigators list. [file BCP-92-2390-s006.pdf]

Appendix S1 – FLARE Investigators list

**Trial Steering Committee**	Kristina Nadrah (Chair)<br>Robert C Read<br>Elizabeth Allen<br>Mahdia Sait
**Independent Data Monitoring Committee**	Stephen Owens (Chair)<br>David Chadwick<br>April Slee<br>Andrew Ustianowski
**UCL Comprehensive Clinical Trials Unit**	Kashfia Chowdhury<br>Felicia Ikeji<br>Amalia Ndoutoumou<br>Hakim-Moulay Dehbi<br>Nick Freemantle<br>Krishneya Anojan<br>Gemma Jones<br>Nazma Begum-Ali<br>Natasha Majid
**Royal Free Hospital Clinical Trials team**	Stephanie Davey<br>Philip Yee<br>Rachel Ochiel<br>Debbie Falconer<br>Stella O'Connor<br>Karl Salazar<br>Tung Le<br>Francesca Gowing<br>Ivy Wanjiku Dakouri<br>Tanaka Ngcozana<br>Sandra Lopez Garces<br>Karima Oduka<br>Daniel Jones<br>Eva Torok-Pollok
**University College London Hospital Clinical Trials team**	Anna Checkley<br>Nicola Longley<br>Michelle Berkeley<br>Esther King<br>Kimberlee Gunn
**Great Ormond Street Hospital laboratories**	Divya Shah<br>Alexander Lennon<br>Abhulya Rai<br>Francis Yongblah<br>Mabel Csatari<br>Kimberly Gilmour
**Royal Free Hospital and UCL (Royal Free campus) laboratories**	Naseem Ahmed<br>Janki Kavi<br>Nimesha Patel<br>Hatim Ebrahim
**University of Birmingham laboratories**	Alex Richter<br>Adrian Shields
